# Supplementary material for: Breakthrough photothermal ammonia decomposition via low-barrier Ni-CeO2-x interfaces on carbon nanotubes
Source: Nat Commun. 2025 Dec 17;16:11433. doi: 10.1038/s41467-025-66325-3 (PMC12749141; doi:10.1038/s41467-025-66325-3)
Supplement: Supplementary file 1 — Supplementary Information [file 41467_2025_66325_MOESM1_ESM.pdf]

Supplementary Information

Breakthrough Photothermal Ammonia Decomposition via Low-Barrier Ni-CeO_{2-x} Interfaces on Carbon Nanotubes

Ruike Tan[1,2], Xiaowei Mu[1,2]*, Xinhui Wang[1,2], Yuxiang Kong[1,2], Qing Ji[1,2], Qingyun Zhan[1,2], Qingchuan Xiong[1,2], Lu Li[1,2]*

[1]College of Chemistry, Jilin University, Changchun 130012, People's Republic of China.
[2]State Key Laboratory of Inorganic Synthesis and Preparative Chemistry, College of Chemistry, Jilin University, Changchun 130012, People's Republic of China.

*Corresponding authors. E-mails: lilymu125@163.com; luli@jlu.edu.cn;

Table of Contents

Supplementary Notes 4
Supplementary Table 1. The ICP-OES measurements of Ni/CNTs, Ni/CeO_2 and Ni-CeO_{2-x}/CNTs. 6
Supplementary Table 2. Comparative properties of CeO_{2-x}/CNTs versus CeO_2. 7
Supplementary Table 3. Summary of Ni-based and Ru-based catalysts with excellent thermal-catalytic performance for ammonia decomposition. 8
Supplementary Table 4. Summary of various reported catalysts in photo- and photo-thermal catalysis for ammonia decomposition. 9
Supplementary Table 5. TRPL decay curve fitting results according to three-exponential decay kinetics. 11
Supplementary Table 6. The distribution of $*NH_x$ (x = 3, 2, 1, 0) surface species for Ni/CeO_2 and Ni-CeO_{2-x}/CNTs after ammonia decomposition via XPS analysis. 12
Supplementary Fig. 1 Powder XRD patterns of a series of samples with varying mass ratios of CeO_2 to CNTs. 13
Supplementary Fig. 2 TEM and HRTEM images of CeO_{2-x}/CNTs (a, b), CeO_2 (c, d) and CNTs (e, f). 14
Supplementary Fig. 3 The N_2 adsorption-desorption isotherms and the pore size distributions of CNTs, CeO_{2-x}/CNTs and CeO_2. 15
Supplementary Fig. 4 Raman spectra of CeO_{2-x}/CNTs and CeO_2. The peak intensity of CeO_2 was reduced by 100 times.. 16
Supplementary Fig. 5 XPS spectra of O 1*s* (a) and Ce 3*d* (b) for CeO_2 and CeO_{2-x}/CNTs. 17
Supplementary Fig. 6 HRTEM images and size distribution of Ni nanoparticles taken from Ni/CeO_2 (a-c), Ni/CNTs (d-f) and Ni-CeO_{2-x}/CNTs (g-i). 18
Supplementary Fig. 7 UPS spectra of CeO_{2-x}/CNTs, CeO_2 and CNTs. 19
Supplementary Fig. 8 Work function of Ni (a), CeO_2 (b), CeO_2 _O_V (c) and CNTs (d). 20
Supplementary Fig. 9 The top and side views of optimal structural models of Ni_8 on CeO_2_O_V (a, c) and CeO_2 (b, d). The Bader charges of each Ni atom in the Ni_8 cluster on different surfaces were presented. Ce: yellow, O: red, Ni: blue, and O_v: green spheres. 21
Supplementary Fig. 10 The top and side views of charge density distribution of Ni_8 on CeO_2_O_V (a) and CeO_2 (b). 22
Supplementary Fig. 11 Schematic diagram of photocatalysis device.. 23
Supplementary Fig. 12 (a) Ni-loaded heterostructured supports composed of CeO_2 and different substances. (b) Ni-loaded heterostructured supports composed of CNTs and different metal oxide. (c) Composition-optimized Ni-CeO_{2-x}/CNTs ternary systems with controlled CeO_2/CNTs mass ratios. (d) CeO_{2-x}/CNTs catalysts loaded with different non-precious metals at fixed 10 wt% active metal content. (e) Ni-CeO_{2-x}/CNTs catalysts with different Ni loadings 24
Supplementary Fig. 13 The evolution curve of the catalyst surface temperature over time under 1.4W cm^{-2} light intensity ($\lambda \geq 400$ nm). 25
Supplementary Fig. 14 Temperature evolution of Ni-CeO_{2-x}/CNTs under the various light intensities ($\lambda \geq 400$ nm). 26
Supplementary Fig. 15 UV-vis DRS of CNTs, CeO_{2-x}/CNTs and CeO_2. 27
Supplementary Fig. 16 Powder XRD patterns for Ni-CeO_{2-x}/CNTs before and after the 55-h stability test (post-test sample mixed with quartz sand) 28

Supplementary Fig. 17 HRTEM images and size distribution of Ni nanoparticles taken from Ni-CeO_{2-x}/CNTs after the 55-hour stability test. 29
Supplementary Fig. 18 EIS Nyquist plots of Ni-CeO_{2-x}/CNTs at different temperatures. 30
Supplementary Fig. 19 The evolution curve of the catalyst surface temperature over time under 920 nm LED irradiation (1.4 W cm^{-2}). 31
Supplementary Fig. 20 Transient photocurrent responses under light irradiation. 32
Supplementary Fig. 21 PL spectroscopy of carriers and their Ni-loaded samples. 33
Supplementary Fig. 22 In situ EPR spectra of Ni-CeO_{2-x}/CNTs under dark, light (10 min), and light with NH_3 co-feed conditions (10 min). 34
Supplementary Fig. 23 The optimized structure of N* on $Ni_8/CeO_2\_O_v$, Ni_8/CeO_2 and Ni(111) with corresponding adsorption energy. 35
Supplementary Fig. 24 *In-situ* XPS spectra of N 1*s* on Ni-CeO_{2-x}/CNTs and Ni/CeO_2 after the ammonia decomposition reaction. 36
Supplementary Fig. 26 Calculation models Optimized geometry of each reaction intermediate for NH_3 decomposition on Ni(111) (a), Ni_8/CeO_2 (b) and $Ni_8/CeO_2\_O_V$ (c).. 39
Supplementary Fig. 27 NH_3 adsorption energy at different sites (a) and the diagrams show the optimized structures of different adsorption sites (b). 40
Supplementary References 41

Supplementary Notes

Calculation of H_2 evolution rate and NH_3 conversion rate

The H_2 evolution rate and NH_3 conversion rate are calculated using the following equation:

$$H_2\ formation\ rate\ (mmol \cdot g_{cat.}{}^{-1} \cdot \min^{-1}) = \frac{Moles\ of\ hydrogenproduced\ (mmol)}{Mass\ of\ the\ Catalyst\ (g) \times Time\ (\min)} \quad (1)$$

$$NH_3\ conversion\ rate\ (\%) = \frac{Moles\ of\ hydrogen\ produced\ (mmol)}{Moles\ of\ ammonia\ \mathrm{int}roduced\ (mmol) \times 1.5} \quad (2)$$

Calculation of the STH efficiency

The solar-to-hydrogen (STH) efficiency was calculated by the following formula (3):

$$\begin{aligned} STH &= \frac{Chemical\ energy\ output}{Solar\ energy\ input} \\ &= \frac{Hydrogen\ production\ rate \times \Delta G_{H_2O \to H_2 + \frac{1}{2}O_2}}{Light\ intensity \times Irradiated\ area} \\ &= \frac{r_{H_2}(mol\ s^{-1}) \times \Delta G_{H_2O \to H_2 + \frac{1}{2}O_2}}{P(W\ cm^{-2}) \times S(cm^2)} \end{aligned} \quad (3)$$

where r_{H2} is the hydrogen production rate, ΔG is the Gibbs energy change (237 kJ mol^{-1}) for water splitting ($H_2O \rightarrow H_2 + 1/2O_2$), P is the incident light intensity and S is the irradiated catalyst area. Under concentrated natural sunlight (8-cm diameter focal spot, 1.08 W·cm^{-2}), the amount of hydrogen produced was 494.76 µmol determined under 1 min natural light irradiation. The specific calculation process is as follows:

$$STH = \frac{\frac{494.76 \times 10^{-6}}{60} mol\ s^{-1} \times 237000\ J\ mol^{-1}}{1.08\ W\ cm^{-2} \times 50\ cm^2} = 3.6\ \%$$

AQE measurement

The Apparent Quantum Efficiency (AQE) was calculated by the following formula[1]:

$$AQE = \frac{n \times Hydrogen\ production\ rate\ (mol \times s^{-1}) \times N_A\ (mol^{-1}) \times Unit\ time\ (s)}{Number\ of\ photons} \quad (4)$$

$$Number\ of\ photons = \frac{Light\ intensity\ (W \times cm^{-2}) \times Irradiated\ area\ (cm^2) \times \lambda\ (m)}{h\ (J \times s) \times c\ (m \times s^{-1})} \times Unit\ time\ (s) \quad (5)$$

where n is the number of electrons transferred per molecule produced. For the production of each hydrogen molecule, two electrons are transferred, which means n is equal to two. N_A represent

Avogadro's constant with values of 6.022×10^{23} mol^{-1}. λ represents the wavelength of the incident light. h is Planck's constant (6.626×10^{-34} J·s) and c is speed of light (3×10^{8} $m\cdot s^{-1}$).

Monochromatic irradiation experiment (400 nm LED) was conducted with controlled photon flux density (1.4 W/cm^2) across a defined irradiation area (4.9 cm^2). Under 5 min irradiation duration, the evolved hydrogen quantity was determined to be 438.03 μmol. The specific calculation process is as follows:

$$AQE = \frac{2\times\frac{438.03\times10^{-6}}{60\times5}\ (mol\times s^{-1})\times6.022\times10^{23}\ (mol^{-1})\times1\ (s)}{\frac{1.4\ (W\times cm^{-2})\times4.9\ (cm^{2})\times400\times10^{-9}\ (m)}{6.626\times10^{-34}\ (J\times s)\times3\times10^{8}\ (m\times s^{-1})}\times1\ (s)} = 12.7\%$$

Computational methods

All density functional theory (DFT) calculations were performed by using the Vienna Ab Initio Simulation Package (VASP, version 5.4.4)[2–4]. The exchange-correlation functional of the generalized gradient approximation with Perdew-Burke-Ernzerhof (GGA-PBE) was adopted in all calculations[5]. The projector-augmented wave (PAW) potentials were used to describe the ion-electron interactions. The cut-off energy was set to 400 eV for the Ni(111) model and 550 eV for all other models[6]. To address the strong electron correlation effects in Ce 4f-orbitals, the Hubbard approximation correction (DFT+U) was applied with an effective Hubbard parameter (U_{eff} =4.5 eV), consistent with previous studies[7–9]. Electronic self-consistency was achieved using a convergence threshold of 10^{-5} eV for energy change, while geometric optimizations were performed until residual forces fell below 0.02 eV/Å. Dispersion interactions were included using the empirical dispersion correction DFT-D3[10]. Transition states for reaction pathways were identified using the climbing image nudged-elastic-band (CI-NEB) method and subsequently validated via vibrational frequency analysis[11]. The charge density difference $\Delta\rho(r)$ was qualitatively evaluated by differential charge density (DCD). The Bader charge analysis was performed for the total charge of each atom in units of unsigned fundamental charge |e|.

The CeO_2 model consisted of $CeO_2(111)$ terminated by oxygen atoms (consisting of 48 Ce atoms and 96 O atoms). Oxygen-deficient $CeO_2(111)$ ($CeO_2\_O_V$) was generated by removing one surface oxygen atom from the pristine model. The CNTs model consisted of a single layer of graphite with 72 C atoms, and the Ni model consisted of Ni(111) slab with 48 Ni atoms. A Ni_8 cluster was adsorbed on each substrate to simulate supported small nanoparticle configurations. All models were modeled with a 15 Å-thick vacuum region along the z-axis.

The adsorption energies of molecule were defined as follows：

$$E_{ads} = E_{total} - E_{surf} - E_{mole} \tag{5}$$

where E_{total}, E_{surf} and E_{mole} are the energies of adsorption configurations, slab models, and free gas molecules, respectively.

Supplementary Tables

Supplementary Table 1. The ICP-OES measurements of Ni/CNTs, Ni/CeO_2 and Ni-CeO_{2-x}/CNTs.

Catalysts	Ni (wt %)	Ni/Ce Molar Ratio	CeO_2/CNTs (wt%)
Ni/CNTs	9.8	N/A	0
Ni/CeO_2	9.7	0.3	N/A
Ni-CeO_{2-x}/CNTs	9.8	0.9	46.9

Supplementary Table 2. Comparative properties of CeO_{2-x}/CNTs versus CeO_2.

Catalysts	$O_{sur}/(O_{lat}+O_{sur}+O_{ads})$ (%)	$Ce^{3+}/(Ce^{3+}+Ce^{4+})$ (%)	I_D/I_{F2g}	BET surface area (m^2/g)
CeO_2	24	31	0.01	33
CeO_{2-x}/CNTs	42	43	0.14	129

Supplementary Table 3. Summary of Ni-based and Ru-based catalysts with excellent thermal-catalytic performance for ammonia decomposition.

Entry	Catalysts	Loading (wt.%)	T (℃)	WHSV ($ml\cdot g_{cat}^{-1}\cdot h^{-1}$)	X_{NH3} (%)	r_{H2} ($mmol_{H2}\cdot g_{cat}^{-1}\cdot min^{-1}$)	Ref.
1	Ni/AlLaCe	20	500	30000	0.59	19.8	15
2	Ni/Y_2O_3-Al_2O_3	48	500	30000	0.61	20.5	16
3	20Ni/La-MgO(5)	20	500	30000	0.63	21.0	17
4	$Ni/Gd_{0.2}Ce_{0.8}O_{2-\delta}$	10	500	30000	0.65	21.8	18
5	Ni-CeO_2(CSCS-3.0)	30	500	30000	0.66	22.2	19
6	$Ni_{1.20}Ce_{0.10}Al$	45	500	30000	0.72	24.1	20
7	Ni/CeO_2	60	500	30000	0.72	24.2	21
8	Ni/MgO@NCFs	10	450	30000	0.99	33.3	22
9	$Ru/La_{0.33}Ce_{0.67}$	1.8	450	30000	0.79	26.5	23
10	$Ru/Ce_5/MgAl_{(600)}$	2.0	450	30000	0.87	27.4	24
11	K-Ru/CNTs	4.8	450	30000	0.97	32.4	25

Supplementary Table 4. Summary of various reported catalysts in photo- and photo-thermal catalysis for ammonia decomposition.

Entry	Catalysts	Ammonia source	Light source	Reactor type	T (℃)	r_{H2} ($mmol_{H2}\cdot g_{cat}^{-1}\cdot min^{-1}$)	Ref.
1	Ce-doped TiO_2	Ammonia solution	UV 8 W Hg pen-ray lamp	Batch reactor	r.t.	1.8×10^{-3}	26
2	rGO/TiO_2 NWs	Ammonia solution	UV 8 W Hg pen-ray lamp	Batch reactor	r.t.	3.5×10^{-3}	27
3	$Pt_{0.9}Au_{0.1}/TiO_2$	Ammonia solution	2000 W Xe lamp	Batch reactor	r.t.	4.7×10^{-3}	28
4	Pt NP/TiO_2	NH_3 with water vapour	300 W Xe lamp	Batch reactor	r.t.	9.3×10^{-3}	29
5	Ni-1.4-MCN	5% NH_3 gas	300 W Xe lamp	Continuous-flow reactor	52	5.9×10^{-4}	30
6	SA Ni/CeO_2	33% NH_3 gas	1 sun	Continuous-flow reactor	310	1.6	31
7	KCC-1-NH_2-Ru@C-K	NH_3 gas	Xe lamp	Continuous-flow reactor	300	5.1	32
8	K-promoted Fe@C	NH_3 gas	300 W Xe lamp	Continuous-flow reactor	250	5.6	33
9	Ru-S-1 (GaOH)	NH_3 gas (99.999%)	400 W Xe lamp	Continuous-flow reactor	400	7.2	34
10	Co@C-ZIF67	NH_3 gas	300 W Xe lamp	Continuous-flow reactor	450	8.0	35
11	Cu-Fe-AR	NH_3 gas (99.99%)	White-light laser	Continuous-flow reactor	352	28.0	36
12	Ru/γ-Al_2O_3	NH_3 gas (99.999%)	300 W Xe lamp	Continuous-flow reactor	418	28.4	37
13	Ru NPs/GaN NWs/Si	Ammonia solution	300 W Xe lamp	Batch reactor	409	184.3	38
14[a]	Ni-CeO_{2-x} /CNTs	NH_3 gas (99.99%)	300 W Xe lamp	Batch reactor	155	22.3	This work
15[b]	Ni-CeO_{2-x} /CNTs	NH_3 gas (99.99%)	300 W Xe lamp	Batch reactor	310	403.8	This work
16[c]	Ni-CeO_{2-x} /CNTs	20% NH_3 gas	300 W Xe lamp	Continuous-flow reactor	152	25.4	This work
17[d]	Ni-CeO_{2-x} /CNTs	NH_3 gas (99.99%)	300 W Xe lamp	Continuous-flow reactor	306	298.4	This work

[a] Batch reaction conditions: Under standard testing conditions, catalytic ammonia conversion (0.5 mmol) was conducted using 5 mg catalyst under light irradiation (300 W Xe lamp, equipped with a 400 nm cut-off filter) with light density maintained at 1.4 W/cm^2, duration = 1 min.

[b] Batch reaction conditions: Catalytic ammonia conversion (0.5 mmol) was conducted using 1 mg catalyst under light irradiation (300 W Xe lamp) with light density maintained at 3.5 W/cm^2, duration = 1 min.
[c] Continuous-flow reaction conditions: A mixture of 5 mg catalyst and 100 mg quartz sand was tested with a 20% NH_3/80% Ar flow rate of 24 mL/min, under light irradiation (300 W Xe lamp, equipped with a 400 nm cut-off filter) with light density maintained at 1.4 W/cm^2.
[d] Continuous-flow reaction conditions: A mixture of 5 mg catalyst and 100 mg quartz sand was tested with a pure NH_3 flow rate of 24 mL/min, under light irradiation (300 W Xe lamp) with light density maintained at 3.5 W/cm^2.

Supplementary Table 5. TRPL decay curve fitting results according to three-exponential decay kinetics.

Sample	τ_1 (ns)	τ_2 (ns)	τ_3 (ns)	Rel_1 (%)	Rel_2 (%)	Rel_3 (%)	χ^2	τ_{ave} (ns)
Ni/CeO_2	0.95	5.30	51.37	11.05	19.83	69.12	0.98	36.66
$Ni\text{-}CeO_{2-x}/CNTs$	1.00	4.63	33.99	25.49	47.44	27.08	1.14	11.65

Supplementary Table 6. The distribution of *NH_x (x = 3, 2, 1, 0) surface species for Ni/CeO_2 and Ni-CeO_{2-x}/CNTs after ammonia decomposition via XPS analysis.

Sample	*NH_3 (%)	*NH_2 (%)	*NH (%)	*N (%)
Ni-CeO_{2-x}/CNTs	37	15	34	15
Ni/CeO_2	18	24	10	48

The N 1*s* spectra were deconvoluted into four peaks: 400.7 eV (*NH_3), 399.7 eV (*NH_2), 398.6 eV (*NH), and 397.4 eV (*N)[39].

Supplementary Figures

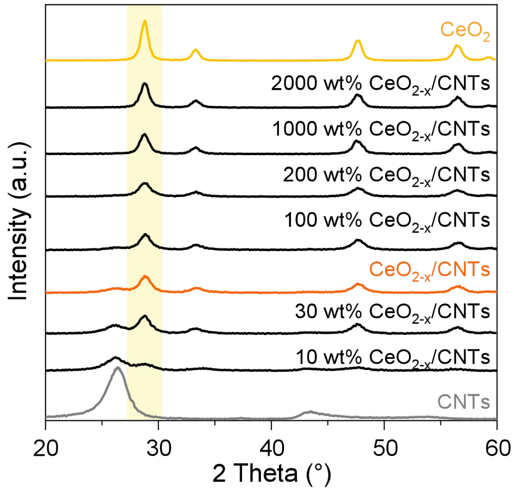

Supplementary Fig. 1 Powder XRD patterns of a series of samples with varying mass ratios of CeO_2 to CNTs.

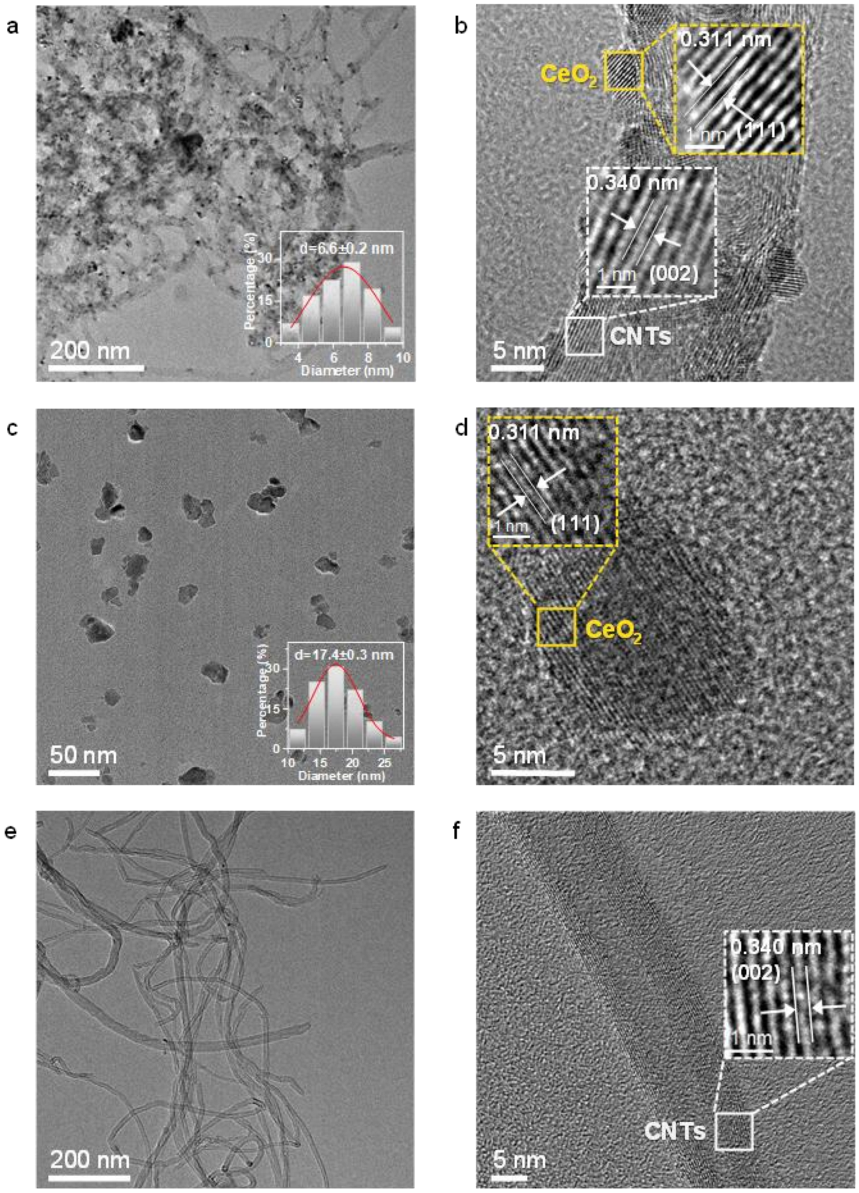

Supplementary Fig. 2 TEM and HRTEM images of CeO_{2-x}/CNTs (a, b), CeO_2 (c, d) and CNTs (e, f).

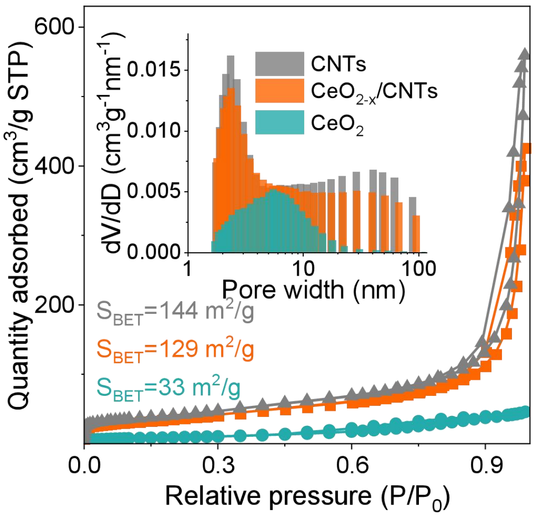

Supplementary Fig. 3 The N_2 adsorption-desorption isotherms and the pore size distributions of CNTs, CeO_{2-x}/CNTs and CeO_2.

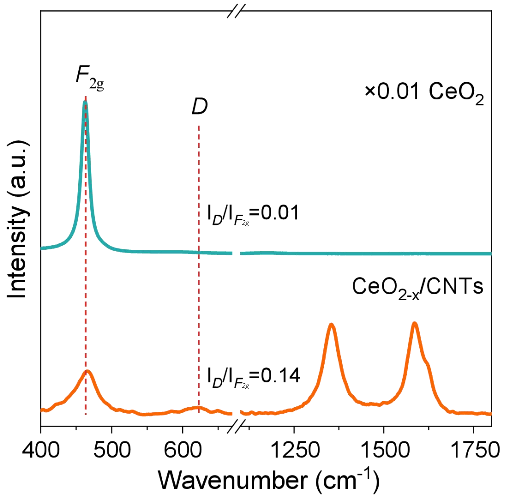

Supplementary Fig. 4 Raman spectra of CeO_{2-x}/CNTs and CeO_2. The peak intensity of CeO_2 was reduced by 100 times. The F_{2g} band at ~467 cm^{-1} is associated with the stretching vibration of the fluorite-type lattice Ce^{4+}-O-Ce^{4+} unit. In addition, the band at ~620 cm^{-1} belongs to the defect-related *D* band, which is caused by oxygen vacancies[12]. The I_D/I_{F2g} peak intensity ratio can be used for semi-quantitative evaluation of oxygen vacancy. The *G* band (~1587 cm^{-1}) and *D* band (~1355 cm^{-1}) are common Raman peaks of carbon materials, representing the in-plane bond stretching mode and the defects of C-C bonds in the hexagonal lattice, respectively[13].

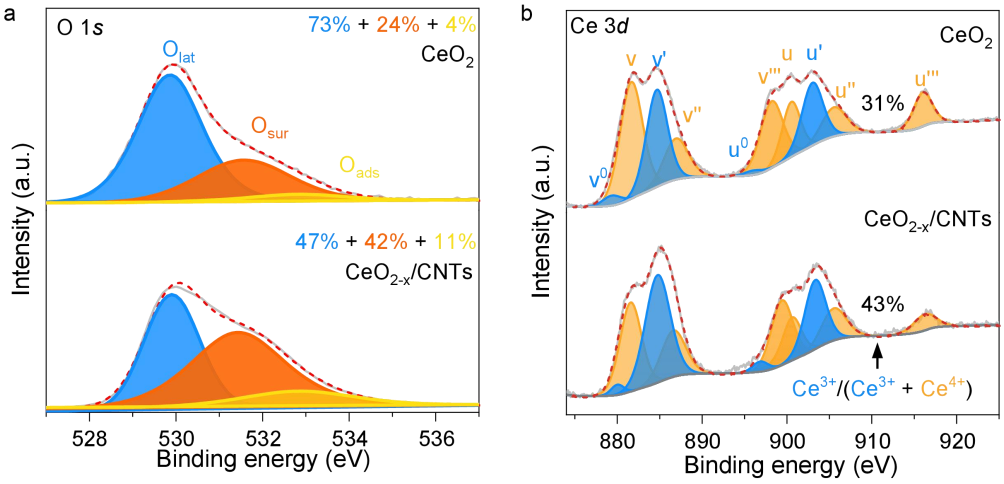

Supplementary Fig. 5 XPS spectra of O 1*s* (a) and Ce 3*d* (b) for CeO_2 and CeO_{2-x}/CNTs. The O 1*s* spectra were deconvoluted into three peaks: 529.9 eV (lattice oxygen, O_{lat}), 531.8 eV (surface oxygen species on oxygen vacancy, O_{sur}), and 533.0 eV (chemisorbed oxygen species, O_{ads}). The ratio of $O_{sur}/(O_{lat} + O_{sur} + O_{ads})$ reflects the concentration of surface oxygen vacancies. The Ce 3*d* spectra can be fitted into ten peaks. Peaks labeled v^0, v', u^0 and u' were assigned to the $3d^{10}4f^1$ state of Ce^{3+} species, while peaks v, v'', v''', u, u'' and u''' were attributed to the $3d^{10}4f^0$ state of Ce^{4+} species[14].

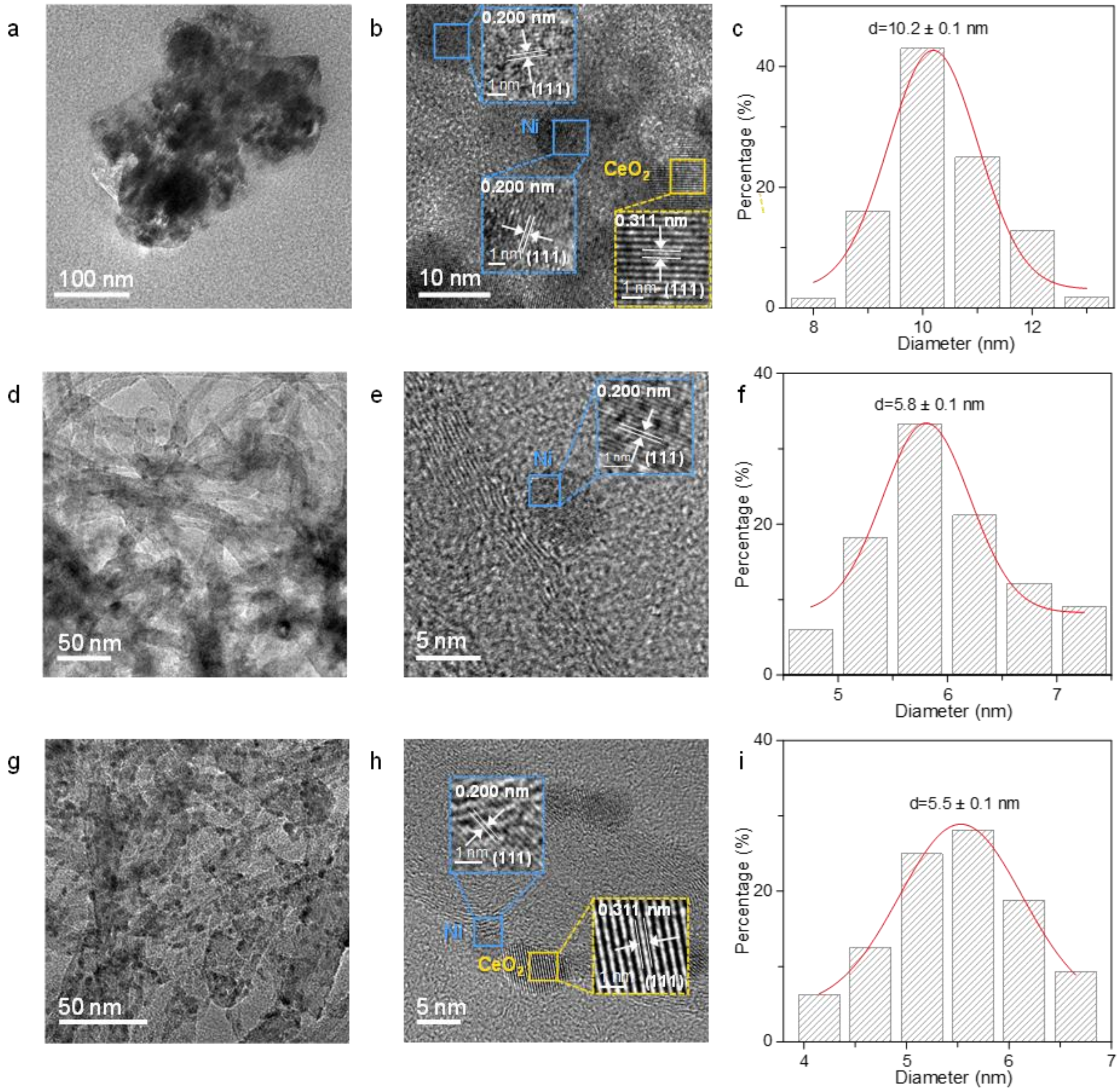

Supplementary Fig. 6 HRTEM images and size distribution of Ni nanoparticles taken from Ni/CeO_2 (a-c), Ni/CNTs (d-f) and Ni-CeO_{2-x}/CNTs (g-i).

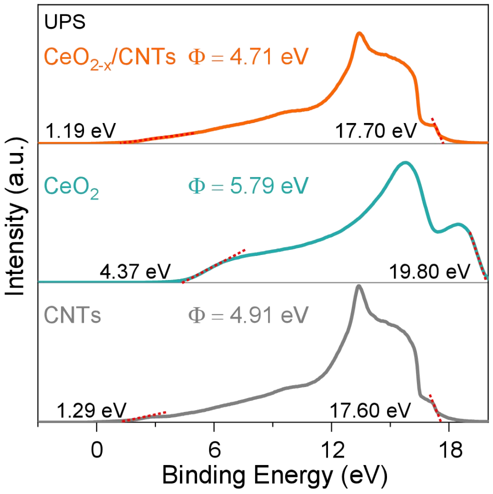

Supplementary Fig. 7 UPS spectra of CeO_{2-x}/CNTs, CeO_2 and CNTs.

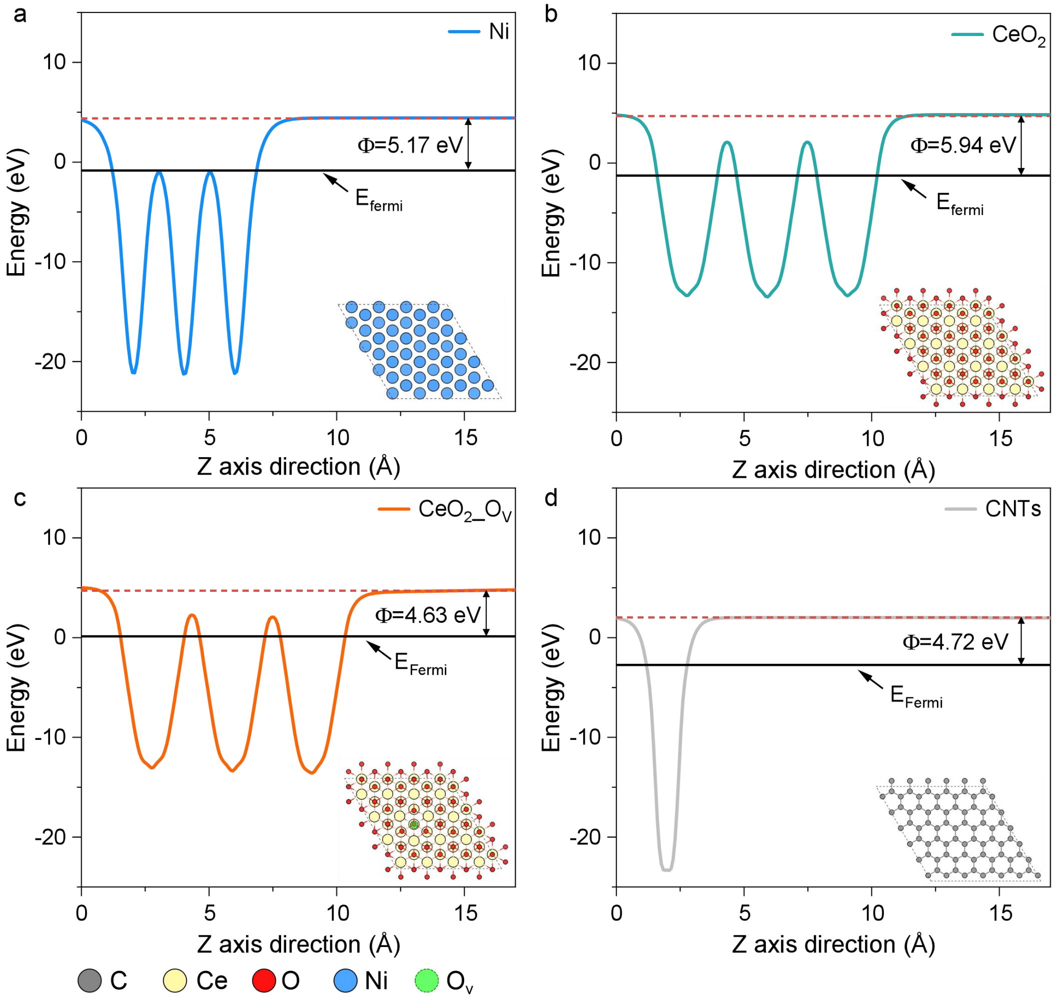

Supplementary Fig. 8 Work function of Ni (a), CeO_2 (b), CeO_2 _O_V (c) and CNTs (d). C: grey, Ce: yellow, O: red, Ni: blue, and O_V: green spheres. For the calculation of the work function, the following expression, $\boldsymbol{\Phi = E_{vac} - E_{Fermi}}$ was used, where $\boldsymbol{E_{vac}}$ was the electrostatic potential energy of the supercell in the vacuum region, and $\boldsymbol{E_{Fermi}}$ was the Fermi energy level.

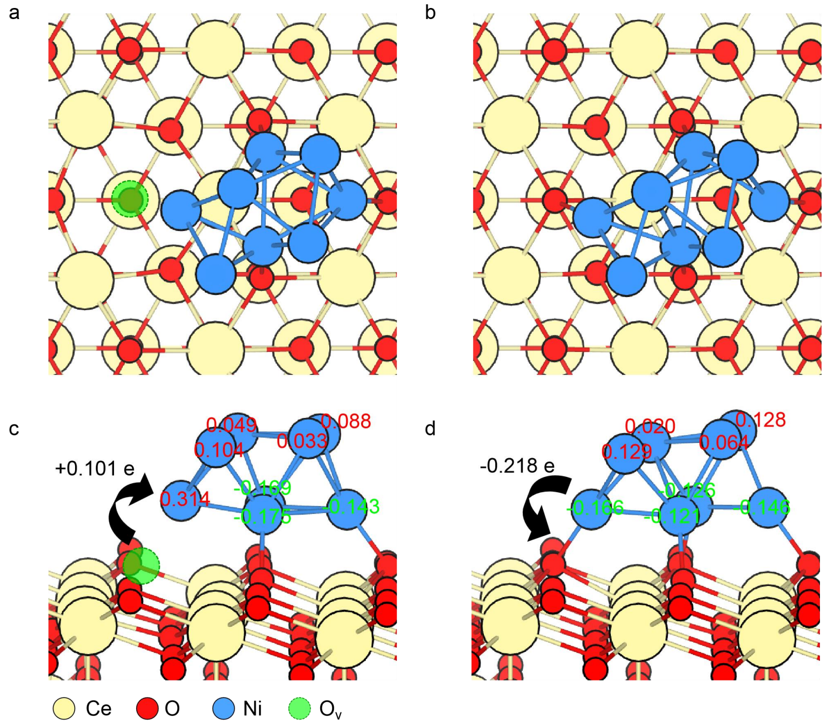

Supplementary Fig. 9 The top and side views of optimal structural models of Ni_8 on CeO_2_O_V (a, c) and CeO_2 (b, d). The Bader charges of each Ni atom in the Ni_8 cluster on different surfaces were presented. Ce: yellow, O: red, Ni: blue, and O_v: green spheres.

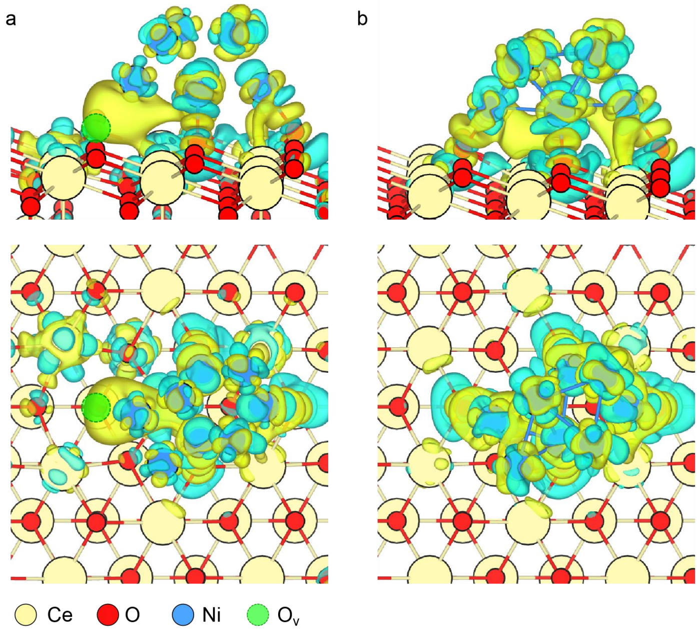

Supplementary Fig. 10 The top and side views of charge density distribution of Ni_8 on CeO_2_O_V (a) and CeO_2 (b). The yellow and cyan regions indicate the gain and loss of electronic charge, respectively. Ce: yellow, O: red, Ni: blue, and O_v: green spheres.

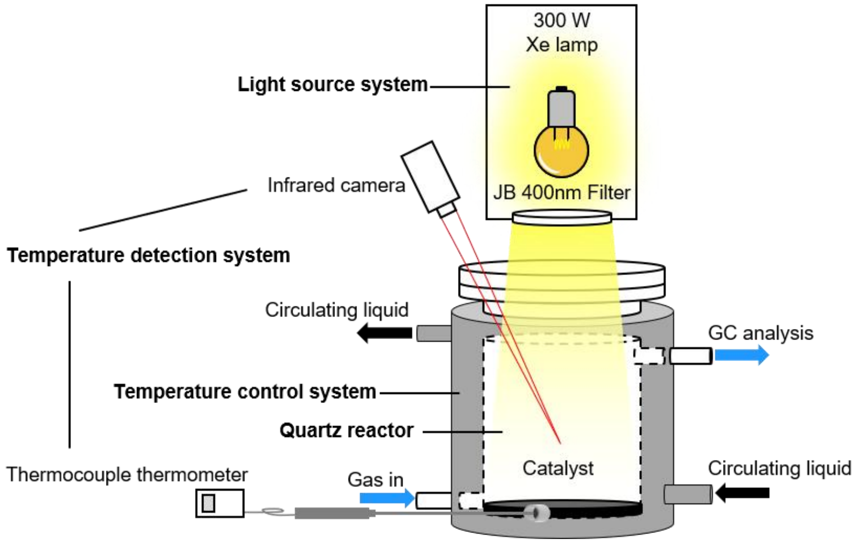

Supplementary Fig. 11 Schematic diagram of photocatalysis device. The device is composed of light source system, temperature detection system, temperature control system, and Quartz reactor.

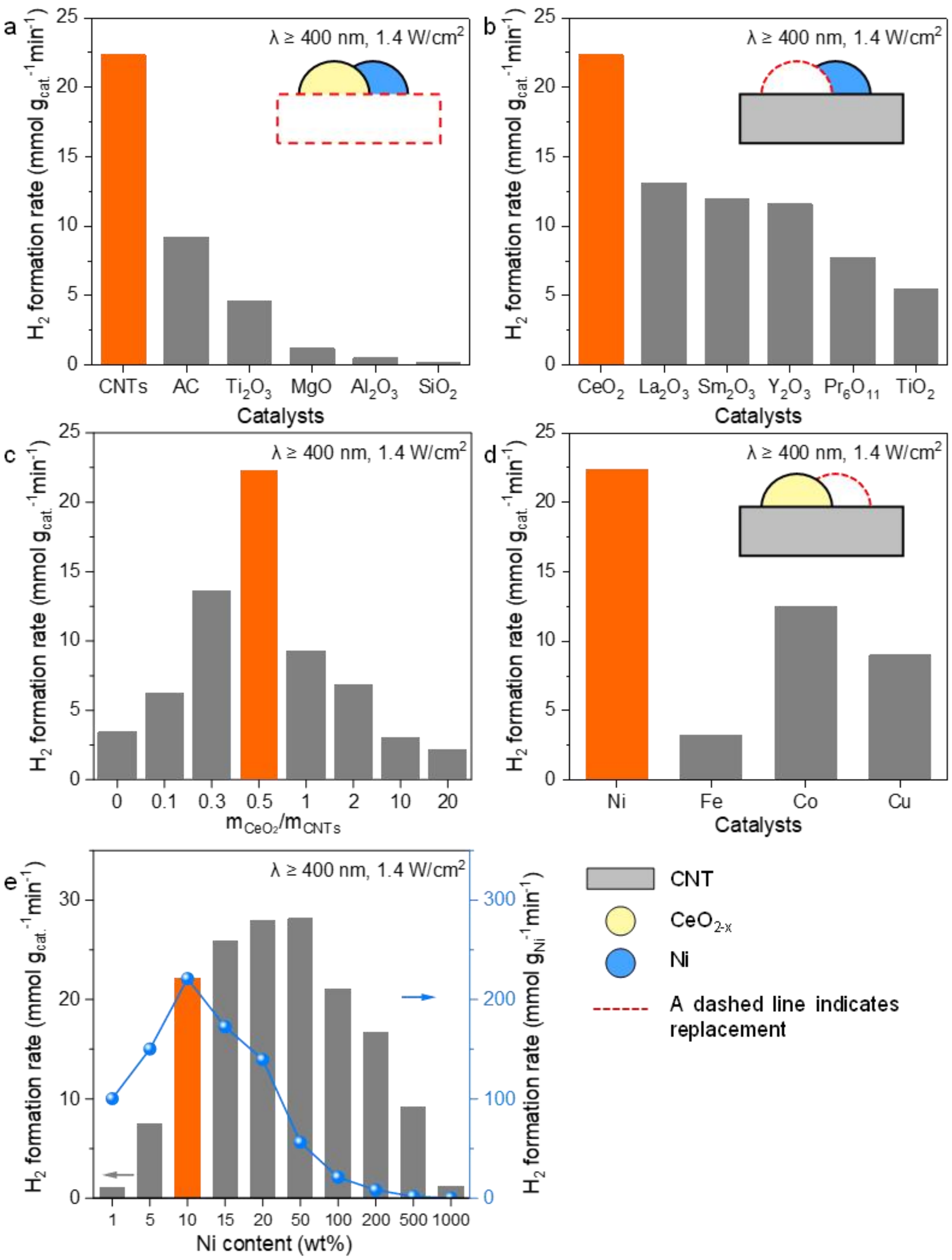

Supplementary Fig. 12 (a) Ni-loaded heterostructured supports composed of CeO_2 and different substances. (b) Ni-loaded heterostructured supports composed of CNTs and different metal oxide. (c) Composition-optimized Ni-CeO_{2-x}/CNTs ternary systems with controlled CeO_2/CNTs mass ratios. (d) CeO_{2-x}/CNTs catalysts loaded with different non-precious metals at fixed 10 wt% active metal content. (e) Ni-CeO_{2-x}/CNTs catalysts with different Ni loadings. Reaction conditions: Under standard testing conditions, catalytic ammonia conversion (0.5 mmol) was conducted using 5 mg catalyst under visible-IR irradiation ($\lambda \geq 400$ nm) with light density maintained at 1.4 W cm^{-2}, duration = 1 min. Schematic representations: CNTs (grey rectangle), CeO_2 (yellow sphere), Ni nanoparticle (blue sphere), Replacement (red dashed lines).

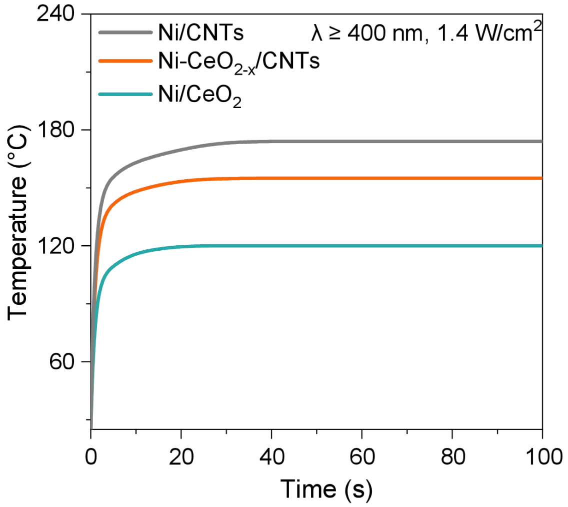

Supplementary Fig. 13 The evolution curve of the catalyst surface temperature over time under 1.4W cm^{-2} light intensity (λ ≥ 400 nm).

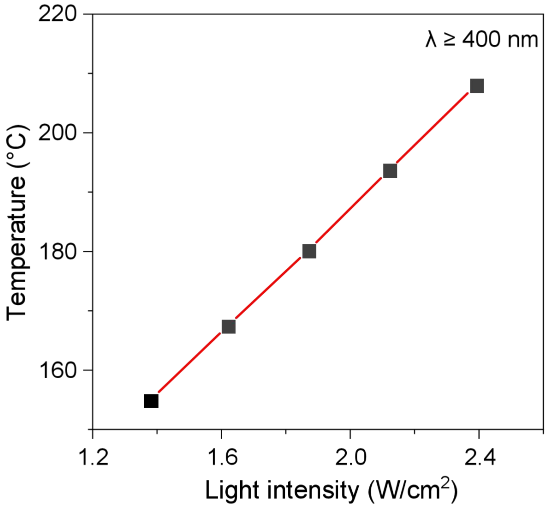

Supplementary Fig. 14 Temperature evolution of Ni-CeO_{2-x}/CNTs under the various light intensities (λ ≥ 400 nm).

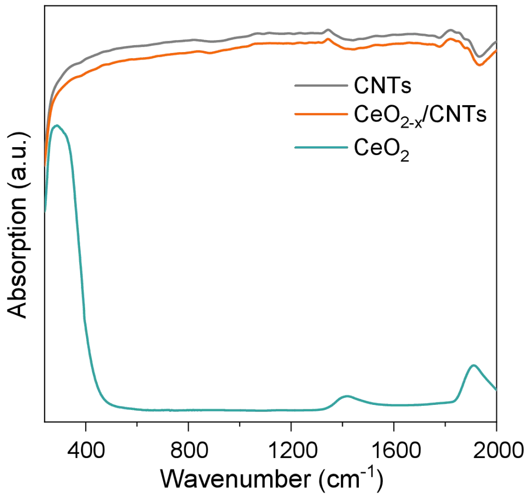

Supplementary Fig. 15 UV-vis DRS of CNTs, CeO_{2-x}/CNTs and CeO_2.

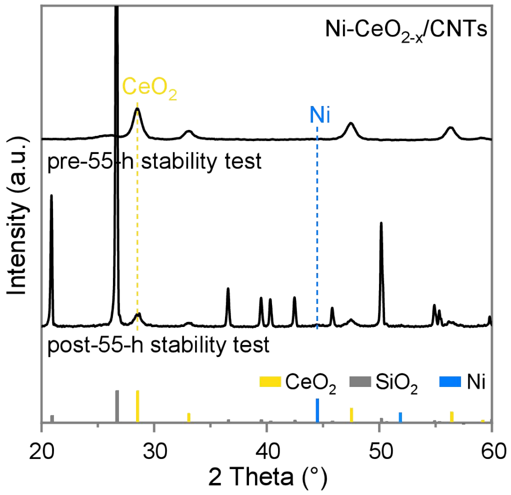

Supplementary Fig. 16 Powder XRD patterns for Ni-CeO_{2-x}/CNTs before and after the 55-h stability test (post-test sample mixed with quartz sand)

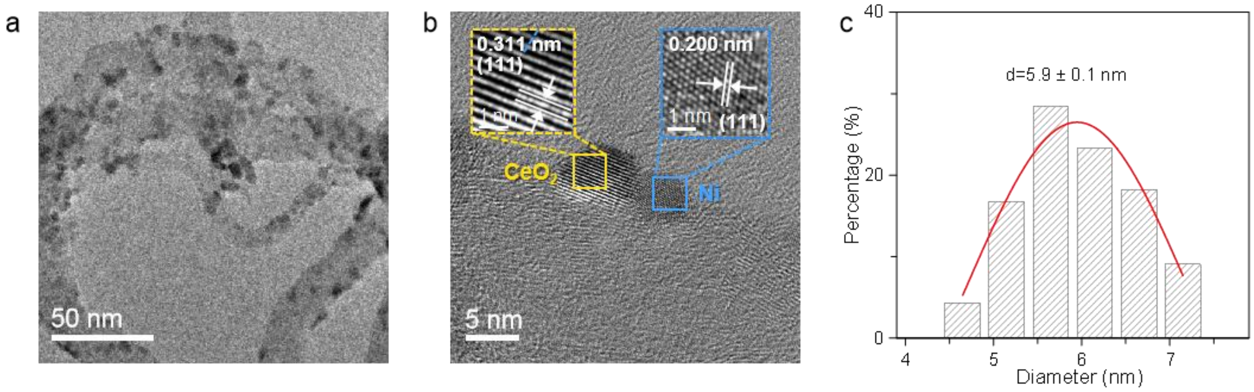

Supplementary Fig. 17 HRTEM images and size distribution of Ni nanoparticles taken from Ni-CeO_{2-x}/CNTs after the 55-hour stability test.

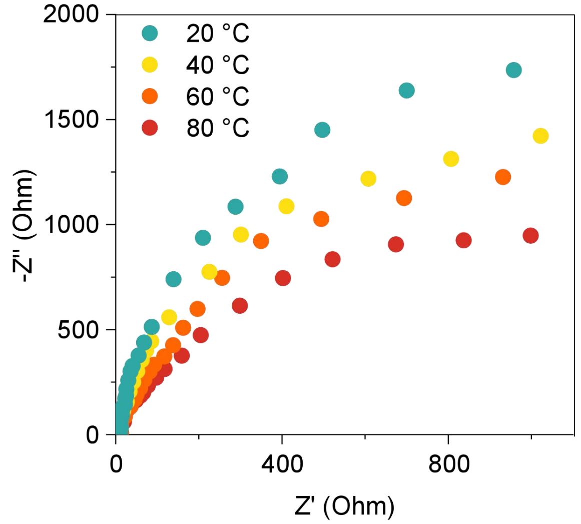

Supplementary Fig. 18 EIS Nyquist plots of Ni-CeO_{2-x}/CNTs at different temperatures. The EIS results show that the Ni-CeO_{2-x}/CNTs sample has the smallest semicircle in Nyquist plots at 80 °C, indicating the lowest charge-transfer resistance.

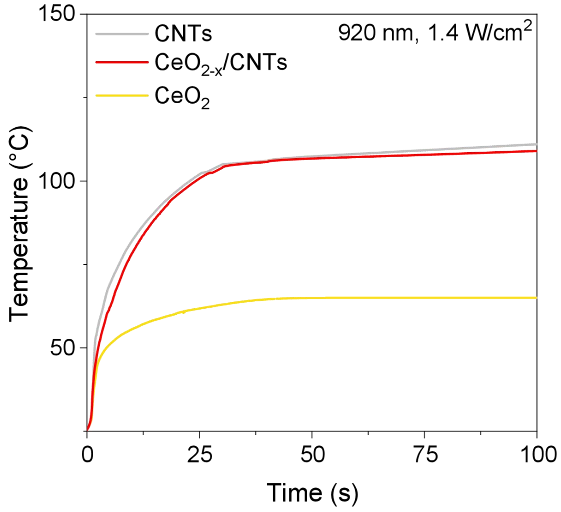

Supplementary Fig. 19 The evolution curve of the catalyst surface temperature over time under 920 nm LED irradiation (1.4 W cm^{-2}).

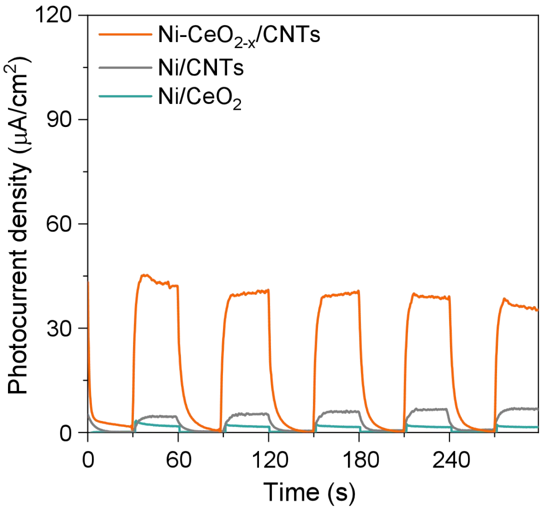

Supplementary Fig. 20 Transient photocurrent responses under light irradiation.

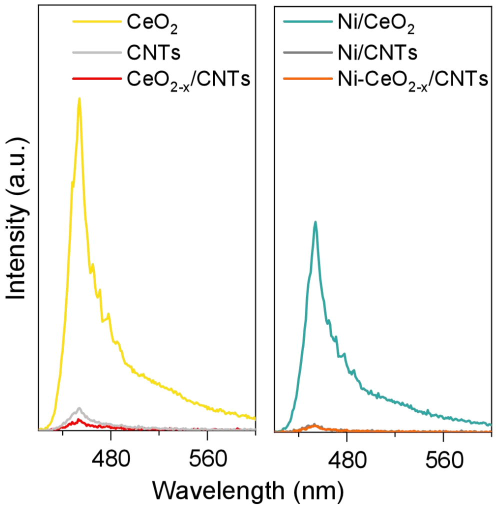

Supplementary Fig. 21 PL spectroscopy of carriers and their Ni-loaded samples.

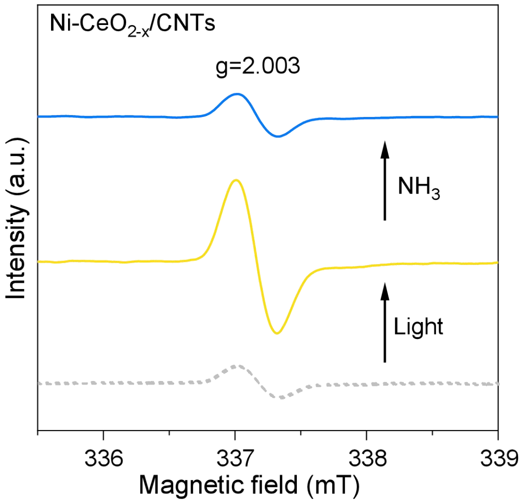

Supplementary Fig. 22 In situ EPR spectra of Ni-CeO_{2-x}/CNTs under dark, light (10 min), and light with NH_3 co-feed conditions (10 min).

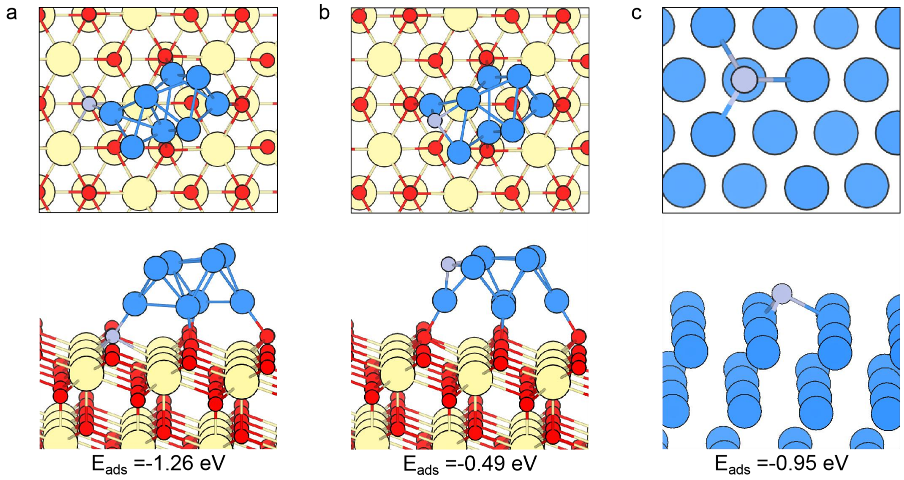

Supplementary Fig. 23 The optimized structure of N* on $Ni_8/CeO_2\_O_v$, Ni_8/CeO_2 and Ni(111) with corresponding adsorption energy. Ce: yellow, O: red, Ni: blue and N: light blue.

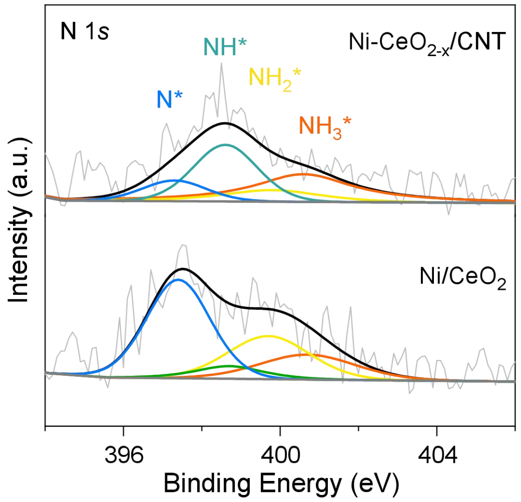

Supplementary Fig. 24 *In-situ* XPS spectra of N 1*s* on Ni-CeO_{2-x}/CNTs and Ni/CeO_2 after the ammonia decomposition reaction.

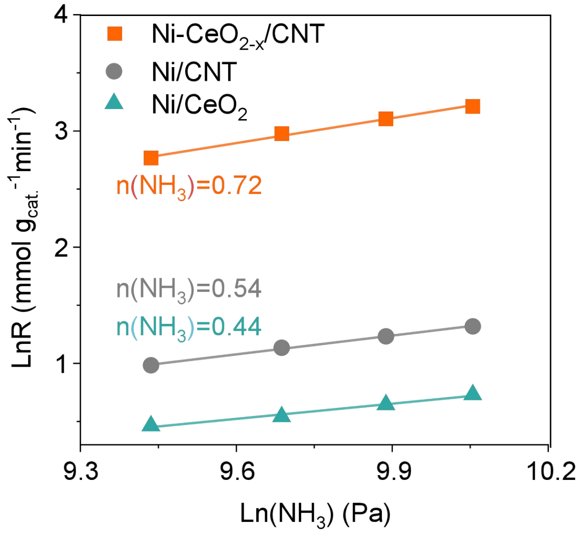

Supplementary Fig. 25 Dependences of NH_3 decomposition rate on the partial pressures of NH_3.

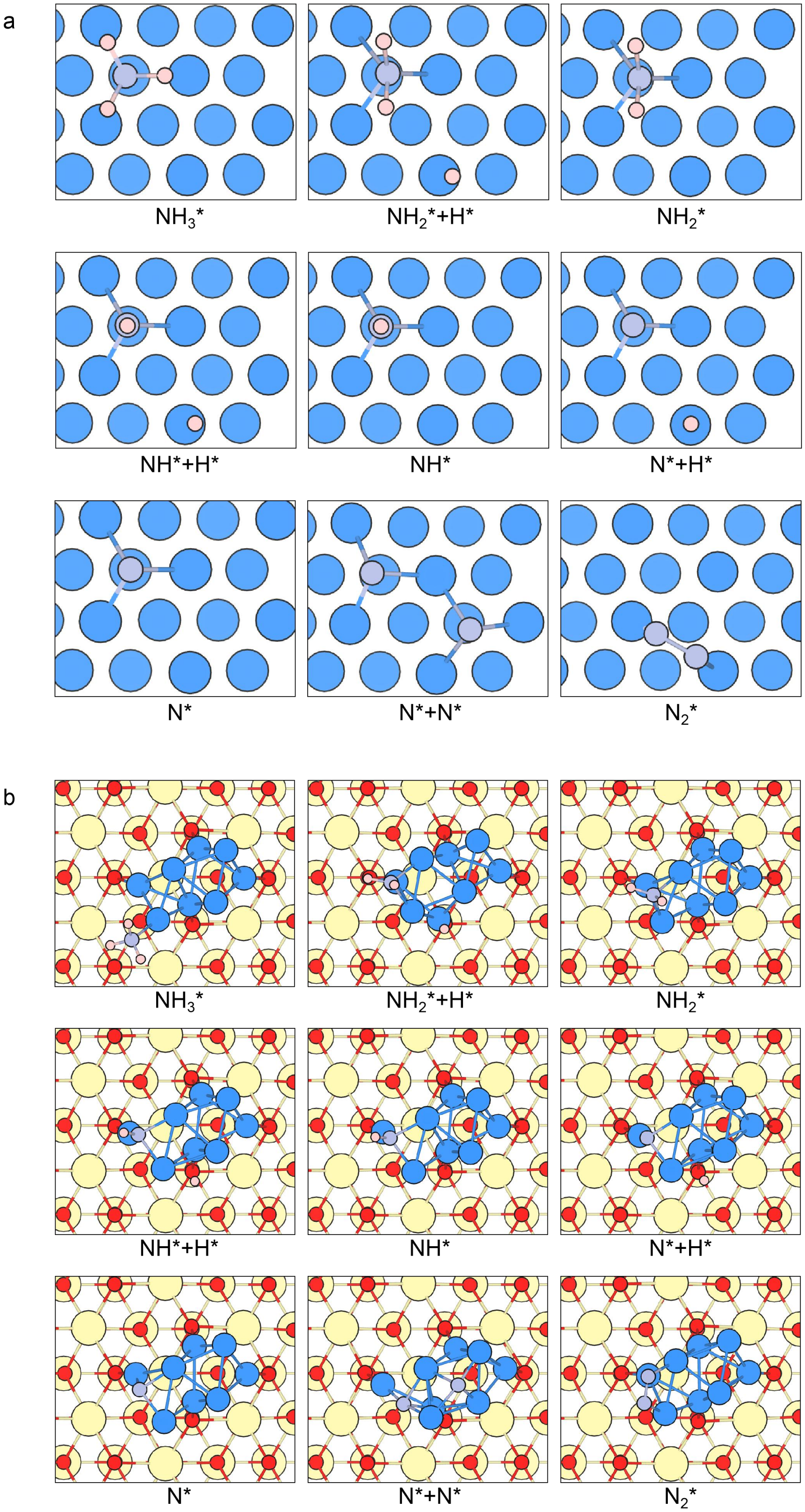
a
NH3*
NH2*+H*
NH2*
NH*+H*
NH*
N*+H*
N*
N*+N*
N2*
b
NH3*
NH2*+H*
NH2*
NH*+H*
NH*
N*+H*
N*
N*+N*
N2*

c

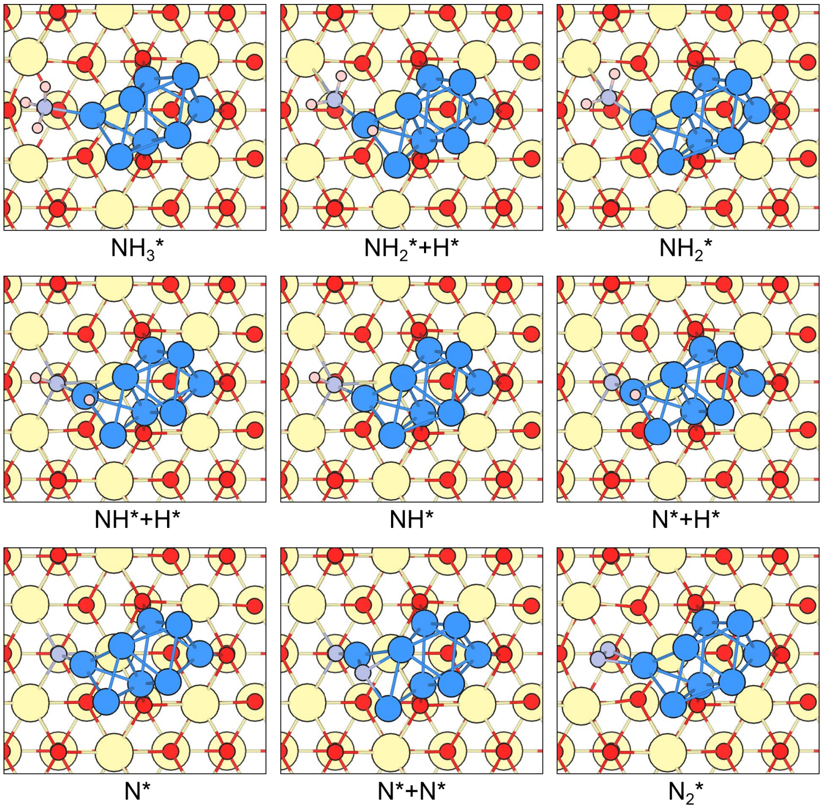

Supplementary Fig. 26 Calculation models Optimized geometry of each reaction intermediate for NH_3 decomposition on Ni(111) (a), Ni_8/CeO_2 (b) and $Ni_8/CeO_2\_O_V$ (c). Ce: yellow, O: red, Ni: blue, N: light blue and H: white.

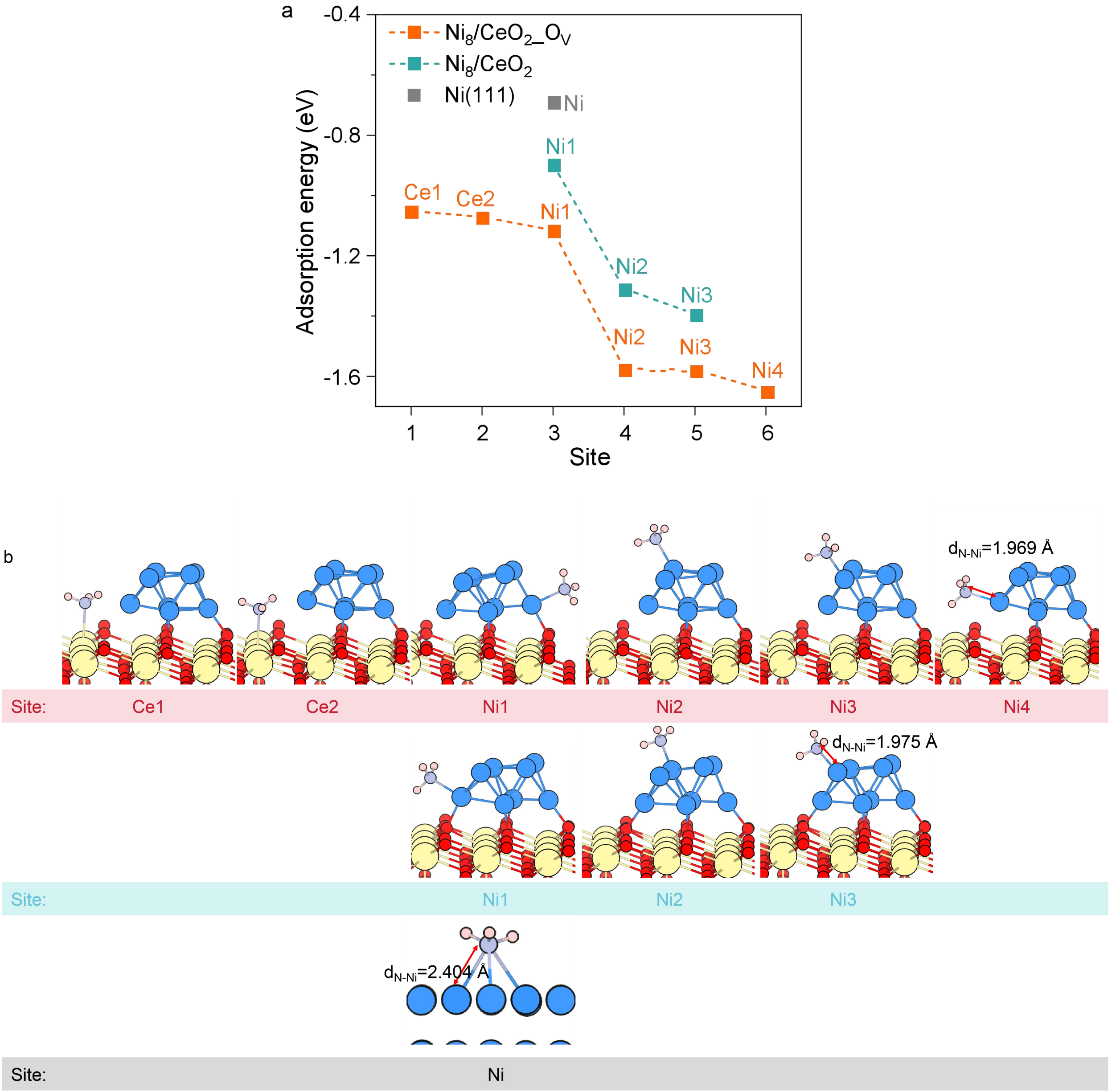

Supplementary Fig. 27 NH_3 adsorption energy at different sites (a) and the diagrams show the optimized structures of different adsorption sites (b). The above, middle and below figures show ammonia adsorption models for $Ni_8/CeO_2\_O_v$, Ni_8/CeO_2 and Ni(111). The optimized structures with the lowest adsorption energy are indicated by the length of the Ni-N bond. Ce: yellow, O: red, Ni: blue, N: light blue and H: white.

Supplementary References

1. Wang, H. *et al.* High quantum efficiency of hydrogen production from methanol aqueous solution with PtCu–TiO_2 photocatalysts. *Nat. Mater.* **22**, 619–626 (2023).
2. Kresse, G. & Furthmüller, J. Efficient iterative schemes for ab initio total-energy calculations using a plane-wave basis set. *Phys. Rev. B* **54**, 11169–11186 (1996).
3. Kohn, W. & Sham, L. J. Self-consistent equations including exchange and correlation effects. *Phys. Rev.* **140**, A1133–A1138 (1965).
4. Hohenberg, P. & Kohn, W. Inhomogeneous electron gas. *Phys. Rev.* **136**, B864–B871 (1964).
5. Jp, P., K, B. & M, E. Generalized gradient approximation made simple. *Phys. Rev. Lett.* **77**, (1996).
6. Blöchl, P. E. Projector augmented-wave method. *Phys. Rev. B* **50**, 17953–17979 (1994).
7. Dudarev, S. L., Botton, G. A., Savrasov, S. Y., Humphreys, C. J. & Sutton, A. P. Electron-energy-loss spectra and the structural stability of nickel oxide: An LSDA+U study. *Phys. Rev. B* **57**, 1505–1509 (1998).
8. Chen, A. *et al.* Structure of the catalytically active copper–ceria interfacial perimeter. *Nat. Catal.* **2**, 334–341 (2019).
9. Fabris, S., de Gironcoli, S., Baroni, S., Vicario, G. & Balducci, G. Taming multiple valency with density functionals: A case study of defective ceria. *Phys. Rev. B* **71**, 041102 (2005).
10. Grimme, S., Antony, J., Ehrlich, S. & Krieg, H. A consistent and accurate ab initio parametrization of density functional dispersion correction (DFT-D) for the 94 elements H-Pu. *J. Chem. Phys.* **132**, 154104 (2010).
11. Henkelman, G., Uberuaga, B. P. & Jónsson, H. A climbing image nudged elastic band method for finding saddle points and minimum energy paths. *J. Chem. Phys.* **113**, 9901–9904 (2000).
12. Nakajima, A., Yoshihara, A. & Ishigame, M. Defect-induced Raman spectra in doped CeO_2. *Phys. Rev. B* **50**, 13297–13307 (1994).
13. Dresselhaus, M. S., Dresselhaus, G., Saito, R. & Jorio, A. Raman spectroscopy of carbon nanotubes. *Phys. Rep.* **409**, 47–99 (2005).
14. Sun, H., Wang, H. & Qu, Z. Construction of CuO/CeO_2 catalysts via the ceria shape effect for selective catalytic oxidation of ammonia. *ACS Catal.* **13**, 1077–1088 (2023).
15. Hu, H. *et al.* Effects of organic compounds on Ni/AlLaCe catalysts for ammonia decomposition to hydrogen. *Ind. Eng. Chem. Res.* **63**, 3910–3920 (2024).
16. Xu, Y.-S., Wang, W.-W., Xu, K., Fu, X.-P. & Jia, C.-J. Multicomponent Ni–Y_2O_3–Al_2O_3 nanospheres as highly efficient catalysts for the ammonia decomposition reaction. *ACS Appl. Nano Mater.* **6**, 19300–19311 (2023).
17. Hu, X.-C. *et al.* Transition metal nanoparticles supported La-promoted MgO as catalysts for hydrogen production via catalytic decomposition of ammonia. *J. Energy Chem.* **38**, 41–49 (2019).
18. He, H. *et al.* Enhanced ammonia decomposition by tuning the support properties of Ni/$Gd_xCe_{1-x}O_{2-\delta}$ at 600 °C. *Molecules* **28**, 2750 (2023).
19. Liu, H., Zhang, Y., Liu, S., Li, S. & Liu, G. Ni-CeO_2 nanocomposite with enhanced metal-support interaction for effective ammonia decomposition to hydrogen. *Chem. Eng. J.* **473**, 145371 (2023).
20. Zheng, W., Zhang, J., Ge, Q., Xu, H. & Li, W. Effects of CeO_2 addition on Ni/Al_2O_3 catalysts for the reaction of ammonia decomposition to hydrogen. *App. Catal. B: Environ.* **80**, 98–105 (2008).

21. Liu, H., Zhang, R., Liu, S. & Liu, G. CeO_2/Ni inverse catalyst as a highly active and stable Ru-free catalyst for ammonia decomposition. *ACS Catal.* **14**, 9927–9939 (2024).
22. Prabu, S., Dharman, R. K., Chiang, K.-Y. & Oh, T. H. Highly efficient Ni nanoparticles embedded on MgO and N-doped carbon nanofibers for efficient ammonia decomposition. *J. Ind. Eng. Chem.* **125**, 402–409 (2023).
23. Le, T. A. *et al.* Ru-supported lanthania-ceria composite as an efficient catalyst for CO_x-free H_2 production from ammonia decomposition. *App. Catal. B: Environ.* **285**, 119831 (2021).
24. Shin, J. *et al.* Elucidating the effect of Ce with abundant surface oxygen vacancies on $MgAl_2O_4$-supported Ru-based catalysts for ammonia decomposition. *App. Catal. B: Environ.* **340**, 123234 (2024).
25. Yin, S. F. *et al.* Carbon nanotubes-supported Ru catalyst for the generation of CO_x-free hydrogen from ammonia. *Catal. Today* **93–95**, 27–38 (2004).
26. Reli, M. *et al.* Novel cerium doped titania catalysts for photocatalytic decomposition of ammonia. *App. Catal. B: Environ.* **178**, 108–116 (2015).
27. Wu, Z. *et al.* Photocatalytic H_2 generation from aqueous ammonia solution using TiO_2 nanowires-intercalated reduced graphene oxide composite membrane under low power UV light. *Emergent Mater.* **2**, 303–311 (2019).
28. Shiraishi, Y., Toi, S., Ichikawa, S. & Hirai, T. Photocatalytic NH_3 splitting on TiO_2 particles decorated with Pt–Au bimetallic alloy nanoparticles. *ACS Appl. Nano Mater.* **3**, 1612–1620 (2020).
29. Yuzawa, H., Mori, T., Itoh, H. & Yoshida, H. Reaction mechanism of ammonia decomposition to nitrogen and hydrogen over metal loaded titanium oxide photocatalyst. *J. Phys. Chem. C* **116**, 4126–4136 (2012).
30. Lin, J. *et al.* Macroporous carbon-nitride-supported transition-metal single-atom catalysts for photocatalytic hydrogen production from ammonia splitting. *ACS Catal.* **13**, 11711–11722 (2023).
31. Li, Y. *et al.* Low Temperature thermal and solar heating carbon-free hydrogen production from ammonia using nickel single atom catalysts. *Adv. Energy Mater.* **12**, 2202459 (2022).
32. Alejandra, R. *et al.* Carbon-coated urchin-like silica nanospheres for enhanced photothermal catalysis. *ChemSusChem* **18**, e202500068(2025).
33. Angel, S. *et al.* Unlocking low-temperature ammonia decomposition via an iron metal-organic framework-derived catalyst under photo-thermal conditions. *Small* **21**, 2411468(2025).
34. Liu, P. *et al.* Hydrogen production from ammonia decomposition catalyzed by Ru nano-particles in alkaline molecular sieves under photothermal conditions. *Mol. Catal.* **543**, 113160 (2023).
35. Angel, S. *et al.* Ammonia decomposition via MOF-derived photothermal catalysts. *ChemSusChem* **18**, e202401896(2024).
36. Yuan, Y. *et al.* Earth-abundant photocatalyst for H_2 generation from NH_3 with light-emitting diode illumination. *Science* **378**, 889–893 (2022).
37. Liu, J. *et al.* Mitigating hydrogen poisoning for robust ammonia-to-hydrogen conversion over photothermal catalysts. *ACS Catal.* **15**, 10470–10479 (2025).
38. Li, J. *et al.* Utilizing full-spectrum sunlight for ammonia decomposition to hydrogen over GaN nanowires-supported Ru nanoparticles on silicon. *Nat. Commun.* **15**, 7393 (2024).
